# Effect of foraminal enlargement on microcrack formation and apical transportation: a nano-CT assessment

**DOI:** 10.1038/s41598-023-31595-8

**Published:** 2023-03-25

**Authors:** Jader Camilo Pinto, Karla de Faria-Vasconcelos, André Ferreira Leite, Mariano Simón Pedano, Juliane Guerreiro-Tanomaru, Reinhilde Jacobs, Mario Tanomaru-Filho

**Affiliations:** 1 Department of Restorative Dentistry, Araraquara Dental School, São Paulo State University (UNESP), School of Dentistry, Rua Humaitá, 1680, Araraquara, SP CEP 14801-903 Brazil; 2 OMFS IMPATH Research Group, Department of Imaging and Pathology, Faculty of Medicine, KU Leuven and Oral and Maxillofacial Surgery, University Hospitals Leuven, Leuven, Belgium; 3 Department of Dentistry, Faculty of Health Sciences, University of Brasília, Brasília, 70910-900 Brazil; 4 Department of Oral Health Sciences, Endodontology and BIOMAT-Biomaterials Research Group, KU Leuven (University of Leuven), UZ Leuven (University Hospitals Leuven), Dentistry, Leuven, Belgium; 5 Department of Dental Medicine, Karolinska Institutet, Stockholm, Sweden

**Keywords:** Oral anatomy, Nanoscale devices

## Abstract

The aim of this study was to evaluate the foraminal enlargement and its influence on microcrack formation and apical transportation in root canals with apical curvature. Eighteen maxillary lateral incisors with apical curvature were selected by using micro-CT images. Root canals were randomly divided in two groups (n = 9) according to root canal preparation using two working lengths: 1 mm short of the apical foramen (control group) and 1 mm beyond the apical foramen (foraminal enlargement). For both groups Reciproc Blue R40 was used for root canal instrumentation. Specimens were scanned by nano-CT (UniTOM HR) before and after root canal preparation. Percentage, length, and width of microcracks, and apical transportation were assessed. Kappa, chi‐square and McNemar tests were used for qualitative analyses while paired and unpaired t-test were used for quantitative analyses (α = 0.05). For both groups, rather similar and low percentages of microcracks were observed before root canal preparation (P > 0.05). The foraminal enlargement promoted new microcracks, not observed in the control group. An increase in microcrack length was observed when the foraminal enlargement was performed (P < 0.05). Higher apical transportation was observed when foraminal enlargement was performed (P < 0.05). Foraminal enlargement using a heat-treated reciprocating file size 40 promoted microcracks and higher apical transportation than root canal preparation up to 1 mm short of apical foramen.

## Introduction

Root canal preparation directly influences long-term endodontic prognosis^1^. However, the apical limit of the root canal instrumentation is still a controversial topic in endodontics^1,2^. Although the most accepted working length is 1 mm short of the apical foramen^1^, the foraminal enlargement technique has also been proposed by some authors^2–4^. This procedure aims to clean the apical foramen^4^ in order to improve the root canal disinfection^2,4^. However, it is still not well defined if foraminal enlargement can promote microcracks and increase transportation of the apical foramen^5–8^.

The presence of dentinal microcracks may influence long-term survival of the tooth^9^, since microorganisms may proliferate in crack lines, leading to the establishment of biofilm on the root surface^10^. In addition, microcracks could potentially promote a vertical root fracture^11^, especially in roots with reduced dentin thickness^12^. Apical transportation may also negatively affect cleaning and filling of the apical root portion, resulting in failure to control infection and to promote root canal sealing^13^.

Micro-computed tomography (micro-CT) has been used to evaluate dentin microcracks, since it is a high precision tool that enables the location of such defects^14^. However, this method has limitations especially for visualizing small microcracks, with lower accuracy than operating microscope with transillumination^15^. Accordingly, to detect small structures or to perform high accuracy measurements, a higher precision tool is required^14^. Nano-computed tomography (nano-CT) devices have small focal spot and increased signal-to-noise ratio, making possible to achieve a maximum spatial resolution in the submicrometer range^16^.

Therefore, the aim of the present study was to evaluate the influence of foraminal enlargement on microcrack formation and apical transportation in maxillary lateral incisors with apical curvature by using nano-CT. The null hypothesis was that the enlargement of apical foramen would not cause microcracks and/or apical transportation.

## Materials and methods

### Ethical implications

The following methods were carried out in accordance with the Declaration of Helsinki and this study was approved by the Research Ethics Committee of the School of Dentistry of Araraquara/UNESP (protocol number: 29320820.8.0000.5416). All teeth used in this study were obtained from the Human Teeth Bank of the School of Dentistry of Araraquara/UNESP.

### Specimen selection

Human maxillary lateral incisors, extracted for reasons not related to this study, were collected. with apical curvature (25°–35°)^17^ previously stored in 0.1% thymol solution were used. The inclusion criteria comprised teeth with complete apical formation, absence of root fractures, calcifications or internal resorptions. Roots were inspected under a stereomicroscope at × 12 magnification to exclude those with any external dentinal defect and roots with immature apices. A digital radiographic system (RVG 6100; Kodak Dental Systems, NY) was used to select teeth according to inclusion criteria. Image J program (National Institutes of Health, Bethesda, MD, USA) was used to assess the degree of curvature in radiographic images. All selected teeth were scanned using a micro-CT device (SkyScan 1276; Bruker-micro-CT, Kontich, Belgium) at a low-resolution (35 µm voxel size). The three-dimensional images allowed a more accurate selection, and the inclusion criteria confirmation, as well as the selection of only teeth that did not present microcracks in the apical third. After exclusion of teeth that did not reach the inclusion criteria, 18 maxillary lateral incisors with apical curvature were selected. Specimens were stored individually in numbered Eppendorf tubes at 100% humidity throughout the execution of the research.

### Root canal preparation

Conventional access cavities were performed, and root canals were explored using a size #10K-file (Dentsply Sirona, Ballaigues, Switzerland). When the tip of the instrument was visible through the main foramen, the working length (WL) was determined. In order to simulate the periodontal ligament, the specimens were assembled in an apparatus with acrylic resin and each tooth was embedded in condensation silicone (Oranwash, Zhermack SpA, Badia Polesine, Italy). The apparatus was immersed in distilled water during the operative steps to prevent dehydration of the specimens. The same dentist, specialist in endodontics, prepared all the teeth.

Lateral incisors were randomly divided into two experimental groups (n = 9), by using a stratified random sampling method, considering the preoperative volume of the root canals. For the control group, a conventional preparation was used, meaning that the WL was defined as 1 mm short of the apical foramen. For the foramen enlargement group, the WL was defined as 1 mm beyond the apical foramen. For both groups, Reciproc Blue R40 (VDW GmbH, Munich, Germany) files were operated by an electric motor VDW SILVER (VDW GmbH) set in the “RECIPROC ALL” function. Each instrument was gradually inserted into the canal using in-and-out movements, at the three levels (cervical, middle and apical) up to WL. Root canal irrigation was performed with 5 mL of 2.5% sodium hypochlorite (NaOCl), using a 30G side-vented needle (NaviTip, Ultradent Products, South Jordan, UT) adapted to a 5 mL syringe (Ultradent Products). As final irrigation, 2 mL of 17% EDTA followed by 5 mL of distilled water were used.

### Nano-CT analysis

For nano-CT image acquisition, a high-resolution protocol (2 µm voxel size) was used to scan all teeth before and after instrumentation by using UniTOM HR device (Tescan, Brno, Czech Republic). A tungsten target was employed, and the voltage and current applied were 65 kV and 123 µA, respectively, with an exposure time of 310 ms, 0.5 mm Al filter and 360° rotation around the vertical axis. Images were reconstructed using Acquila reconstruction software (Acquila v.2900), and superimposed with geometric alignment by DataViewer software program (Data Viewer v.1.5.1, Bruker). Quantitative and qualitative analyzes were performed using a CTAn software program (CTAn v.1.14.4, Bruker). To create the three-dimensional images, the CTVox software (CTVox v.3.2; Bruker) was used.

Analyses were performed in the 2-mm apical region of the roots. For qualitative analyses of microcracks, cross-sectional images before and after instrumentation were simultaneously assessed. Then, these were compared by two blinded examiners at two different time points. Microcracks were defined as lines or defects extending from inside the root canal to the dentin or from the outer root surface into the dentin. The distribution of microcracks was expressed as a percentage of the total cross-sectional images^18^. The qualitative analyses of microcracks were based on their length and width. For length measurement, the extension of slices containing the microcrack was recorded, knowing that each slice represents exactly 0.002 mm, the number of slices was multiplicated for this value, and the result was considered as the microcrack length in mm. For width measurement, the extension of the microcracks was measured in each 10 cross-sectional slices, corresponding to 0.020 mm.

Measurement of the apical transportation were obtained before and after instrumentation on cross-sectional images of the roots. As proposed by Gambill et al.^19^, the following formula was used to calculate the root canal transportation: (X1–X2) − (Y1–Y2). X1 represented the shortest distance from the outside of the curved root to the periphery of the canal before instrumentation, Y1 represented the shortest distance from the inside of the curved root to the periphery of the canal before instrumentation, X2 represented the shortest distance from the outside of the curved root to the periphery of the instrumented canal, and Y2 represented the shortest distance from the inside of the curved root to the periphery of the instrumented canal. Ten cross-sectional images, determined by the arithmetical mean value, were measured in the last 2 mm of apex of each root.

### Statistical analysis

For sample calculation, G* Power 3.1.7 for Windows program (Heinrich-Heine-Universität Dusseldorf, Dusseldorf, Germany) was used. Chi‐square and t test were used with an Alpha type error of 0.05 and Beta power of 0.95. Previous studies were used to determine the specific effect size for percentage of microcracks, 0.93^20^; and root canal transportation, 3.11^21^. A total of 8 specimens was indicated as being the ideal size required.

All outcome data were analyzed with the GraphPad Prism 7.00 statistical software package (GraphPad Software, La Jolla, CA, USA).

For qualitative analyses of microcracks, results were expressed as the percentage for each group. Kappa statistics was used for intra- and inter-examiner agreement. McNemar test was used to determine significant differences before and after instrumentation and chi‐square test was used for comparations between groups. For the qualitative analysis of microcracks and transportation, the normality of the data was tested using the Shapiro–Wilk test. The paired t-test was used for comparisons between before and after instrumentation. For comparisons between the groups non-paired t test was used. The level of significance was 5% for all the analysis.

## Results

High intra and inter-examiner agreement was found with Kappa values higher than 0.91 for all evaluations.

**Figure 1:**
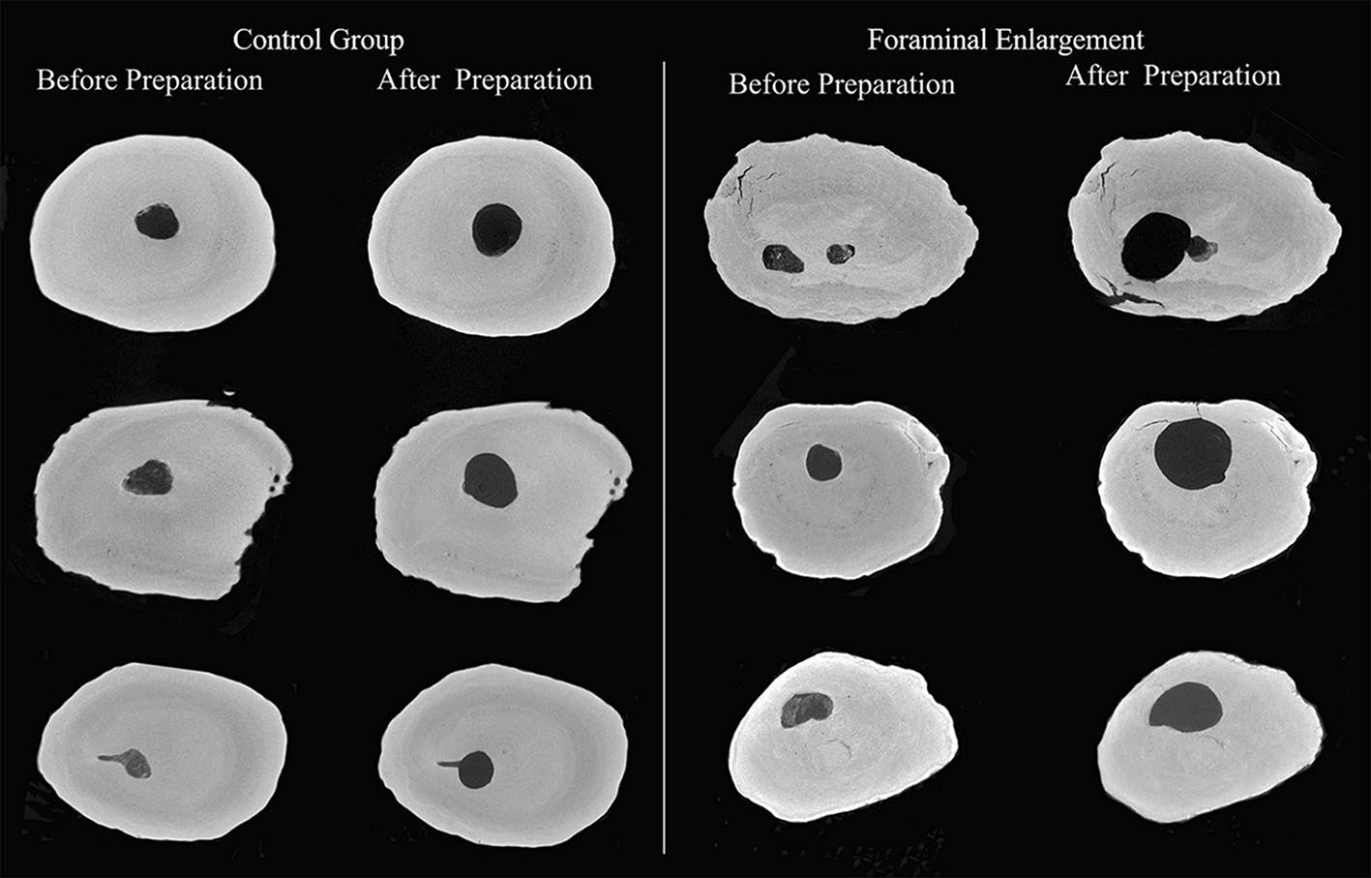
Representative cross-sectional nano-CT images showing the formation of microcracks after instrumentation for apical enlargement group.

Table 1 shows the mean of slices presenting microcracks, taking into account all evaluated samples. These results indicate a low percentage of microcracks before preparation, being similar between groups (P > 0.05). The enlargement of apical foramina caused new microcracks with a higher percentage when compared to the preparation 1 mm short of apical foramen (P < 0.05). New microcracks were not found in the control group (P > 0.05). Figure 1 shows the appearance of new microcracks only after foraminal enlargement, as this finding was not detected in the control group.

**Table 1:** Percentage of dentinal microcracks before and after preparation.

	Before preparation	After preparation
Control group	3.83^aA^	4.40^aA^
Foraminal enlargement	3.73^aA^	12.87^bB^

Different lowercase letters overlapped on the same line indicate statistical difference between before and after preparation in same group. Different capital letters overlapped on the same column indicate statistical difference between groups.

Table 2 indicates the mean values and standard deviations of the length and width of microcracks observed before and after instrumentation. An increase in the microcracks length could be observed after foraminal enlargement (P < 0.05), while in the control group the length values were similar before and after instrumentation (P > 0.05). The width of cracks was similar between groups and before and after instrumentation for both groups (P > 0.05).

**Table 2:** Mean and standard deviation of the length and width (mm) of the microcracks found before and after preparation.

		Before preparation	After preparation
Length	Control group	0.23 ± 0.06	0.23 ± 0.08
	Foraminal enlargement	0.22 ± 0.04^a^	0.33 ± 0.07^a^
Width	Control group	0.23 ± 0.07	0.27 ± 0.08
	Foraminal enlargement	0.26 ± 0.04	0.27 ± 0.05

^a^Represents statistical difference between before and after preparation.

**Figure 2:**
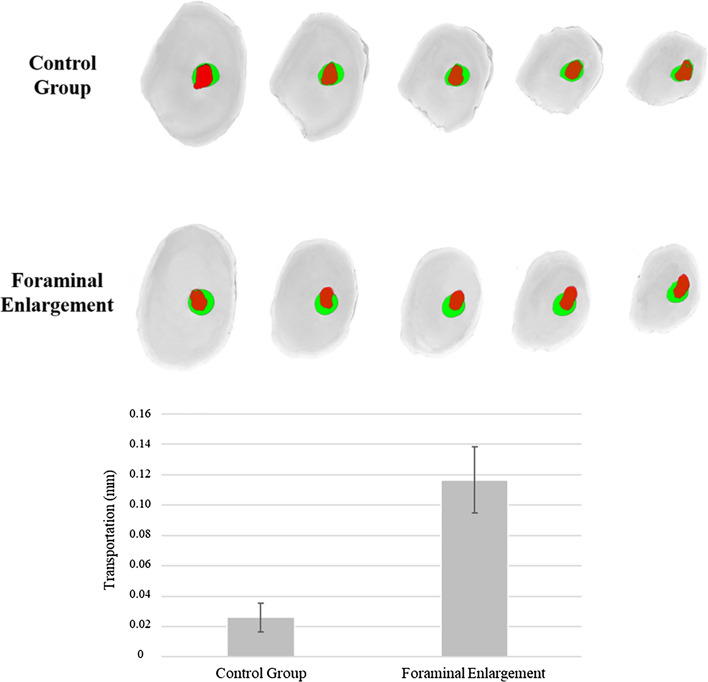
Representative cross-sectional of 3D reconstructions showing superposition of the root canal before instrumentation (red) with after instrumentation (green) and bar chart showing mean and standard deviation of the transportation (mm) caused in the root canals after preparation.

**Figure 3:**
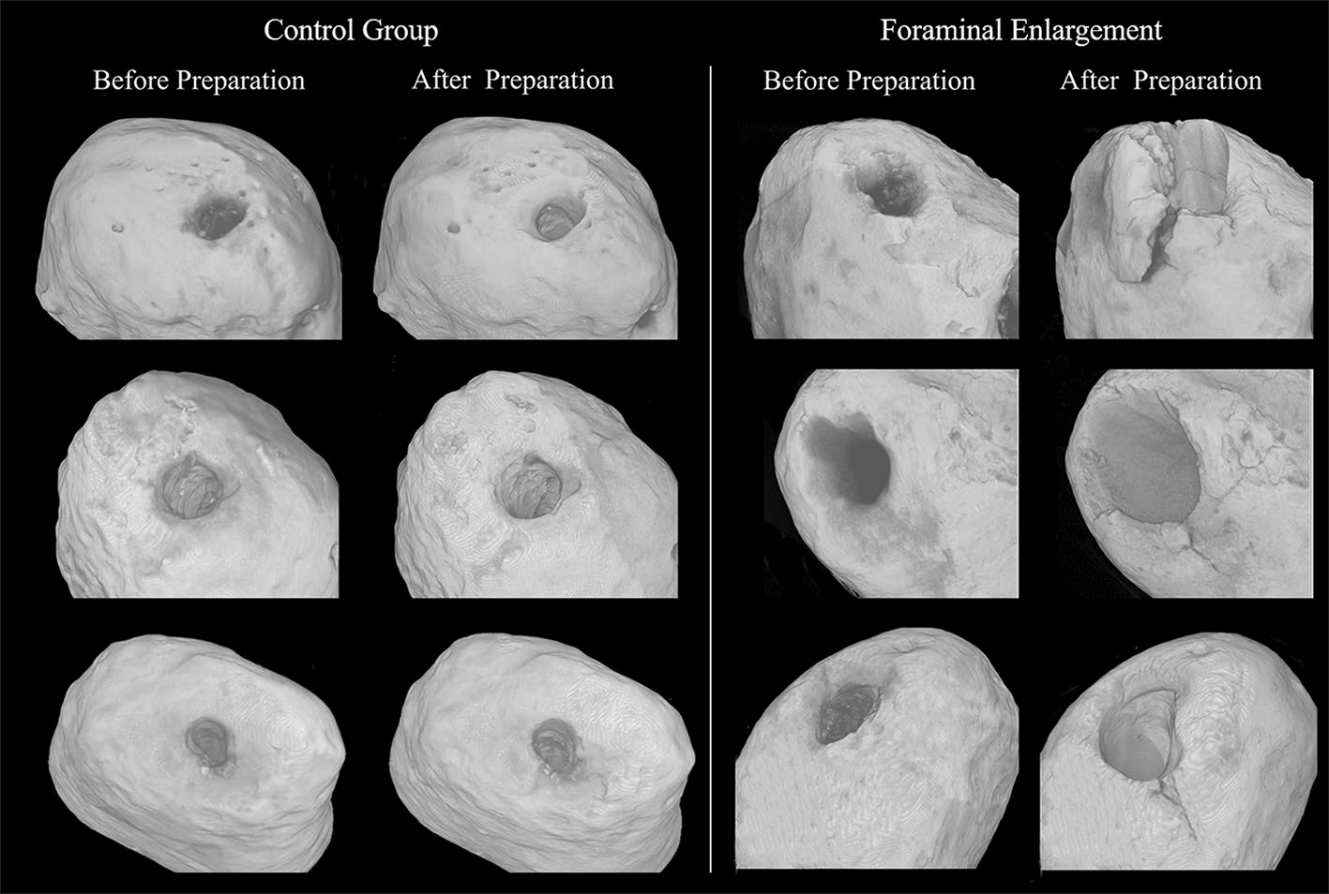
3D reconstruction of nano-CT showing the presence of microcracks and apical deformations after foraminal enlargement.

Figure 2 indicates significant higher apical transportation after foramen enlargement in comparison with the control group (P < 0.05). Figure 3 shows the presence of microcracks and apical deformations in 3D reconstructed images of nano-CT.

## Discussion

The results of this ex-vivo study suggest that the enlargement of apical foramen using a heat-treated reciprocating file size #40 promoted the formation of microcracks and apical transportation. Therefore, the null hypothesis was rejected.

The current literature is still controversial regarding whether the limit of root canal preparation affect treatment prognosis^1,2^. Moreover, a lack of consistency is observed in relation to the association between the working length and the presence of microcracks^5–8^^.^ These conflicting results may be attributed to the use of different methodological designs, teeth morphology and analytical methods^15^. Therefore, this is the first study to assess the influence of working length on microcrack formation and on root foramen transportation by using nano-CT system.

A recent study did not find any correlation between the working length and the formation of new dentinal microcracks after preparation, however with a different methodological approach, especially regarding the size of apical preparation^8^. In this previous study, a lower apical preparation was performed until the size 0.25 mm by using Reciproc Blue R25. Nevertheless, the use of larger apical preparation has been recommended to improve the effect of irrigating solution^22^, and to enhance root canal disinfection^23^. In addition, the current state-of-art of the heat-treated NiTi instruments, transversal sections and tapers results in safer root canal preparation of curved root canals^24^. The aforementioned statements justify the use of a larger apical preparation with the reciprocating NiTi file Reciproc Blue R40 in the present study. On the other hand, the higher size of preparation may have influenced microcrack formation after foraminal enlargement.

During sample selection, micro-CT images were used to select only those teeth that did not have microcracks. However, the high-resolution nano-CT images allowed accurate location of the microcracks before and after root canal preparation. In both groups, the microcracks presented small size, achieving an average length of 0.22 mm and an average width of 0.27 mm. When the foraminal enlargement was performed, microcracks were longer after preparation, while in the control group, preoperative microcracks remained unchanged. These microcracks can be a potential site for bacterial infection^6^, significantly decreasing the long-term prognosis of the teeth.

In both groups, a small percentage of microcracks were observed before root canal instrumentation, as also observed in previous studies^25–28^. These microcracks can be attributed to the forces during extraction and excessive occlusal functional loads or even tooth age^29^. Dentin dehydration during experimental steps also have been referred as a risk factor for microcracks appearance^30^. However, to prevent the dentin dehydration the teeth were stored in 0.1% thymol and during nano-CT scans the specimens were covered with parafilm paper. Additionally, besides the facts that each specimen has acted as its own control, in the control group no new microcracks were found after instrumentation.

The foraminal enlargement caused more transportation in the root apex than the instrumentation performed 1 mm short of apical foramen. Reciproc Blue files are submitted to a heat treatment that improves their flexibility^31^, making them suitable for instrumentation of curved root canal^32^. However, previous studies have shown some transportation, even using instruments with heat treatment^8,33^. Some authors have also pointed out that this transportation can occur more frequently in preparations 1 mm beyond the foramen^8,34^. Root canal transportation may negatively affect the outcomes of endodontic treatments^35^, especially in the apical region that represents a critical zone to maintain infection of the root canal system^36^. In addition, as can been observed in Fig. 3, the transportation is still associated with deformations of the apical foramen^8^. In agreement, a previous study also observed higher apical foramen deformations after foraminal enlargement^34^. Moreover, it should be emphasized that such deformations may affect the sealing of the root canal filling.

Even with the inherent limitations related to an ex-vivo investigation, our results support the concept of restricting the root canal instrumentation procedures to the root canal limit, especially when larger files are used. Although some authors have recommended the enlargement of apical foramen to improve the apical cleaning^37^, this might increase microcrack formation and canal transportation. Further studies are necessary to verify the influence of foraminal enlargement on filling adaption as well on the root canal cleaning.

## Conclusion

In this ex-vivo study, the evaluation of ultra-resolution images showed a higher number of microcracks and apical transportation with the foraminal enlargement technique using heat-treated reciprocating files when compared to conventional preparation until 1 mm short of apical foramen.

## Data Availability

The datasets used and analyzed during the current study are available from the corresponding author on reasonable request.
